# Genomics England’s implementation of its public engagement strategy: Blurred boundaries between engagement for the United Kingdom’s 100,000 Genomes project and the need for public support

**DOI:** 10.1177/0963662517747200

**Published:** 2017-12-14

**Authors:** Gabrielle Natalie Samuel, Bobbie Farsides

**Affiliations:** Brighton and Sussex Medical School, UK; King’s College London, UK; Brighton and Sussex Medical School, UK

**Keywords:** ethics, genomics, public engagement, science communication, whole genome sequencing

## Abstract

The United Kingdom’s 100,000 Genomes Project has the aim of sequencing 100,000 genomes from National Health Service patients such that whole genome sequencing becomes routine clinical practice. It also has a research-focused goal to provide data for scientific discovery. Genomics England is the limited company established by the Department of Health to deliver the project. As an innovative scientific/clinical venture, it is interesting to consider how Genomics England positions itself in relation to public engagement activities. We set out to explore how individuals working at, or associated with, Genomics England enacted public engagement in practice. Our findings show that individuals offered a narrative in which public engagement performed more than one function. On one side, public engagement was seen as ‘good practice’. On the other, public engagement was presented as core to the project’s success – needed to encourage involvement and ultimately recruitment. We discuss the implications of this in this article.

## 1. Introduction

In 2009, and as part of the long political push to implement genomics into healthcare, the UK House of Lords Science and Technology Committee called for the development of a ‘*strategic vision for genomic medicine in the UK*’.^1^ In response, the Conservative government established the Human Genomics Strategy Group designed to monitor advances in genomics and develop a vision for the discipline within the National Health Service (NHS). The committee’s January 2012 report explained the various steps needed to streamline genomics within the NHS and as such laid the foundations of the United Kingdom’s 100,000 Genomes project (Human Genomics Strategy Group, 2012: 9). In December 2012, the 100,000 Genomes Project was launched with the advertised aim of sequencing 100,000 genomes of NHS patients, focusing on rare disease, cancer and infectious disease. Its central goal was to implement genomics innovation/testing on a national scale such that it becomes routine in NHS practice. Alongside this, it also had a research-focused goal to provide data for scientific discovery, making it the first ever research-clinical hybrid project within the NHS.^2^ The project received over £200m in initial investment (Monitor Deloitte, 2015) and gained specific support from the UK Prime Minister David Cameron whose eldest son had been born with a genetic condition (Summers and Sparrow, 2009).

Genomics England is the limited company wholly owned by the Department of Health tasked with carrying out the rare disease and cancer arm of the 100,000 Genomes Project. In describing its remit, the company states four central aims: to bring benefit to patients, to create an ethical and transparent programme based on consent, to enable new scientific discovery and medical insights and to kick-start the development of a UK genomics industry.^3^ Thus, while the project was born out of a desire to bring patient benefit, to improve NHS infrastructure and to drive research in the genetics arena, as with many biotechnologies, it was also ‘*justified in terms of [its] potential to generate economic value*’ and as such exemplifies the ‘bioeconomy’ at play (Petersen and Krisjansen, 2015: 30). Economic opportunities are anticipated from services required for DNA sequencing, data analysis and clinical interpretation. Investment is anticipated in areas of analytics, data management and ethics training (Monitor Deloitte, 2015). The 100,000 Genomes Project is therefore much more than a DNA sequencing project, and although clearly embedded within the NHS, its impact and influence stretch far beyond, and many different stakeholders will be involved in its activities.

At the same time as the advancement of genomic medicine was being pursued, policy makers and healthcare providers were becoming increasingly aware of the need for public engagement around matters of science and medicine (Stilgoe et al., 2014). In light of a growing academic literature (Burchell et al., 2009; Etchegary et al., 2015; O’Doherty and Burgess, 2009; Secko et al., 2008), universities and other research organisations were also becoming increasingly convinced of the need to engage with the public, in part to evidence the public value of their work as required by the research impact agenda (Martin, 2011; Owen et al., 2016). As an innovative scientific/clinical venture involving partnership between politicians, policy makers, patients, clinicians, scientists and commercial companies, it is interesting to consider how Genomics England positioned itself in relation to this increasingly important activity.

The influential National Co-ordinating Centre for Public Engagement (n.d.) defines public engagement as ‘*the myriad of ways in which the activity and benefits of higher education and research can be shared with the public*’ . Pivotal to public engagement strategies is a two-way dialogue between specialists and the public so that specialists can listen to the public, develop their understanding of their views and incorporate those views within their practices (Higher Education Funding Council for England, 2006; Select Committee on Science and Technology, 2000). However, debate still continues in terms of identifying what does and does not qualify as public engagement, and the exact approach taken by any particular organisation will depend on their brief and target audience (Pieczka and Escobar, 2013). Avard et al. (2010) note, ‘*there is confusion about what is meant by public involvement, as it can have many different meanings along a continuum that ranges from low levels of communication to higher levels of involvement*’ (p. 511). Allen et al. (2014) also note there is

confusion as to how public engagement should occur, the appropriate degree of involvement, the goals that involvement should seek to achieve, whom ‘the public’ includes, what methods of involvement should be used, and how the success and utility of involvement should be assessed. (p. 13)

This is not surprising given the US National Academy of Sciences’ recent 2016 report *Communicating Science Effectively*, which notes that science communication is complex, context and audience specific (National Academies of Sciences, Engineering and Medicine, 2016).

In contrast, Pieczka argues that it is sometimes difficult to avoid a ‘one-size fits all’ approach to public engagement which risks it being seen as a means for achieving *all* institutional goals, especially in organisations which have a number of different remits all of which somehow relate to the public. Public engagement then becomes ‘*a technology to educate publics, legitimize investments, improve public relations, manage risk, and deal with the media*’ (Pieczka and Escobar, 2013: 121). For example, in a recent study, van Bekkum and colleagues identified a variety of ways in which 10 UK-based non-commercial funding bodies either partially or exclusively funding health or medical research, and explicitly promoting public engagement, interpreted and implemented their role to promote public engagement. Here, notions of public engagement were being interplayed with those of science communication and selling/promotion (Van Bekkum et al., 2016)

It would therefore appear that many public engagement initiatives borrow from the traditional, influential though largely discredited deficit model of public understanding of science in which, rather than ‘engaging’, the task at hand is to educate a deficient public in order to (hopefully) build trust in science. Indeed, many institutions and cultural practices have been shown to still implicitly embody, or reinvent, notions of the deficit model within their public interaction activities (Brunk, 2006; Wynne, 2006).

Against this backdrop, we set out to explore how individuals working at, or associated with, Genomics England approached their public engagement responsibilities. This work was conducted as part of a speculative study of the ethical issues arising for those working on the 100,00 Genomes Project with a view to proposing a larger study which will now be taking place under the aegis of a large collaborative award.

In terms of policy, Genomics England is publicly committed to notions of public engagement: ‘transparency’ is one of the organisation’s four central aims, and a statement of their approach to public engagement appears on the Genomics England’s website: ‘*[we are] working with patients, patient groups and charities, patient and public involvement (PPI) groups and participants throughout Genomics England and the 100,000 Genomes Project. We also have a programme of public events and debates*’. However, it is well established that policymaking can be distinct from what happens at the level of practice (Greenhalgh et al., 2004; Peters et al., 2013), and, as we have shown above, this is likely compounded for public engagement initiatives – especially in complex organisations. This is because of the pluralistic definitions regarding what constitutes public engagement, and the therefore blurred boundaries that may exist between the need to engage with the public, versus the need for other modes of communication such as those related to promotion. We have called this distinction the theory–practice gap in relation to public engagement.

We wished to explore the theory–practice gap in Genomics England’s public engagement strategy. Both the literature and Genomics England’s policy commitment do not reflect on such a gap, but rather assume a clear role for public engagement such that its practice is protected from any association with ethically fraught issues such as recruitment, information provision or consent in a research setting. We set out to empirically investigate what was happening in practice and therefore contribute to the growing literature on the theory–practice gap in public engagement. Initial findings of our study were encouraging, highlighting an active engagement strategy within the company and demonstrating an acknowledgment of the heterogeneity and complexity of different populations within public and patient communities. Alongside this, however, there was also a clear understanding of the organisational need to garner public and patient support for the project in order to drive recruitment, and thus ensure the project’s success clinically, scientifically and politically. This meant that people offered a narrative in which public engagement performed more than one function, and in which the possibility of conflicting goals for public engagement were apparent – although this was not always explicitly recognised. On one hand, public engagement was seen as ‘good practice’, providing information about genomics and the 100,000 Genomes Project and creating a two-way dialogue in which Genomics England listened to and collaborated with patients and the public about their concerns with the project. On the other hand, public engagement was presented as core to the project’s success – building public commitment to the project by encouraging involvement and ultimately recruitment. This was sometimes, though not always, seen to perform a function similar to traditional practices around the public understanding of science. These findings point to a theory–practice gap in public engagement, and our findings contribute to this literature. We discuss the findings in more detail below.

## 2. Methods

As a medical ethicist and a medical sociologist working in the field of ethics, we were particularly interested in exploring how the ethical issues associated with the 100,000 Genomes Project have been, and are being, addressed. Second author, Bobbie Farsides (BF), is an unpaid member of Genomics England’s Ethics Advisory Committee (EAC), which was established to ensure that Genomics England’s strategy and policy decisions were subject to independent ethical scrutiny, and as such has a high level of understanding of the machinery behind the 100,000 Genomes Project and the ethical governance structures in place (Samuel and Farsides, forthcoming). Her experience and knowledge of Genomics England played a role in conducting this speculative study and also allowed first author, Gabrielle Samuel (GS), to identify a number of on-going issues pertinent to her interviews with members of the organisation. During these interviews, while many ethical issues were discussed, interviewees also spoke at length about Genomics England’s public engagement strategy. It is this aspect of the interviews which is reported here.

While BF is affiliated with the EAC, she has a long history of researching the practice of healthcare professionals and scientists working in ethically challenging fields, and has worked for many years with GS on the UK Wellcome Trust-funded LABTEC project, which explored such practices. During this time, both BF and GS have reflected upon their ability to work closely with those they research while remaining independent in their research and analysis.

### Recruitment

In order to contact those working at or associated with Genomics England, we sought permission from Board Member and EAC Chair, Professor Mike Parker, who BF was acquainted with, given her role on the EAC. After viewing and sharing the project’s proposed rationale and methodology with Genomics England, Mike granted permission for the project to proceed. First author, GS, spoke with Head of Ethics, Laura Riley, about who it would be best to interview to ensure a full range of opinions, stakeholders and institutions was garnered about the 100,000 Genomes Project and Genomics England. Laura Riley had access to names and email addresses of the relevant individuals who covered the diverse range of stakeholders the authors wished to speak to, which she kindly forwarded to us. While this could be described as a convenience sample, all major actors in this small organisation were represented by interview (see below) and by the completion of the interviews, data saturation had been reached.

Neither Mike Parker nor Laura Riley were interviewed for this project. Moreover, they nor Genomics England, more broadly, had any knowledge of who responded to the recruitment emails (and hence who participated in the project). They also had no input into the project design, interview questions which were asked, nor the analysis of findings.

Potential respondents were recruited in the summer of 2016, at the approximate half-way mark through the 100,000 Genomes Project. Invites requesting participation in the research study, including participant information sheets, were emailed to 37 individuals associated with or those who worked for Genomics England. Individuals from the following categories were invited: Genomics England staff members (including those involved in public engagement); Genomics England board members; EAC members; representatives from the Department of Health, Public Health England, NHS England and Genomics Medicine Centres (GMCs);^4^ and those involved in the evaluation of the 100,000 Genomes Project. Individuals were requested to respond to the email invite if they were interested in participating in the research project, or if they had any questions. A maximum of two follow-up emails were sent to non-responding individuals.

### Interviews

A total of 20 individuals responded to the email invite and 20 semi-structured interviews were conducted. All of the categories of individuals listed in the above section were represented in the interviews (exact numbers are not provided to protect confidentiality). All participants signed a consent form prior to the interviews commencing. Interviews were conducted either by telephone or face-to-face (at a location chosen by the participant), lasted between 30 and 105 minutes, and were recorded. The interview schedule was broad, asking participants about their background and their role associated with the 100,000 Genomes Project. Participants were also asked their views on the project, on its benefits (present and potential) and drawbacks, on any issues they had come across in relation to their role in the project and how these had been overcome, and on the project’s ethics and public engagement strategy. Interviews were transcribed either by GS or by an external transcribing service.

### Analysis

Analysis of interview data was approached using inductive reasoning employing the inductive approach of grounded theory (Charmaz, 2006; Strauss, 1987). The analysis (or coding) of data was based on two inter-linked rounds: overview analysis and detailed analysis (Strauss, 1987). Overview analysis consisted of memo-making and broad coding. Extensive memo-making was employed by the interviewer directly after each interview. Broad coding proceeded by scanning the interview transcripts for relevant ideas and themes. Detailed analysis of the full transcripts occurred line-by-line using NVivo software. Coding was carried out via constant comparison, which was continual, rigorous and allowed for developing and refining of conceptual categories as theory was developed. Due to the limited number of individuals associated with Genomics England, and the need to protect confidentiality population, comparisons between respondents from different institutions are not reported.

## 3. Findings

Interviewees provided a wide variety of examples of public engagement strategies implemented by Genomics England. These included meetings in town halls and other settings aimed to gauge the publics’ attitude to genomics; focus groups which explored (potential) participant’s views on the project; the establishment of patient and participant panels within the NHS; collaborative events with, for example, Cancer Research UK and the United Kingdom’s Wellcome Trust; and commissioning public engagement type exercises to start a public conversation about genomics and explore public understanding and public perceptions of issues around the project. The Genomics England website was also viewed as a useful platform for explaining and providing information about the 100,000 Genomes Project.

At the heart of all of these strategies were notions of public engagement which aligned closely with those reported in the academic literature (National Co-ordinating Centre for Public Engagement, n.d.). Genomics England representatives expressed a genuine desire to listen to the public and patients, to pay attention to their views and to concomitantly alter their approach in light of these views (‘*all our decisions were influenced by feedback we got from the research that was done*’ (Interviewee 17)). Interviewees provided examples to explain this. For instance, one interviewee described how, in response to public concerns, original plans to store genomic information in a ‘cloud’ had been scrapped in place of a more physical storage facility:

to give you a really strong piece of evidence of how we listen to people, at the outset of the project they gave us a strong steer that they didn’t want their data in a cloud run by companies like Amazon or Google. So we have a fixed data centre. (Interviewee 16)

In a second example, Genomics England had made the decision to invite a number of project participants to join the Genomics England data access committee – a committee which controls access to the data and samples collected within the project, be that by academic researchers, clinicians or industry partners. In this way, they responded to concerns expressed by participants regarding access to their genomic data:

The access review committee is chaired by a scientist and there are two or three scientists on it. But there’s also four lay people on it. And these are … representatives of participants, the idea being that if you’re enrolled in the program, you should be sitting there when people request for access to data saying, ‘actually I enrolled in this program and I’d actually like these people to be working on this data’. Or ‘Actually, I’m not sure I like what they’re proposing; perhaps they can look at it and come back to us with a better proposal’. Or ‘Indeed, this is on your list of unacceptable uses and I don’t want my data used this way’. So we’ve put them at the heart of the decision-making process in terms of access to the data. (Interviewee 16)

Openness and transparency were seen as a crucial component of Genomics England’s public engagement strategy (‘*what was required was an “honest[y] about stuff”*’ (Interviewee 14)). Interviewees offered various reasons for why they perceived openness and transparency to be so important. For some, transparency was an intrinsic ethical responsibility, ‘*we’re very transparent about what will happen to data. We’re very transparent about storing the data..[..]..that’s ethically responsible*’ (Interviewee 16). This was important because the project was ‘working with public money’: *‘I don’t think working with public money … we should do things that we need to hide … it’s a principle underpinning everything I do*’ (Interviewee 4). For others, transparency seemed to be crucial because of more extrinsic reasons such as supporting patient recruitment.^5^ Some felt that public engagement was the best way to counter misleading and/or potentially damaging ideas about the organisation, with interviewees worried about the ‘*danger [that] you can have a perfectly ethical project but it’s portrayed in a way to patients, that they misunderstand what’s happening*’ (Interviewee 7). Interviewees were concerned that public and patient misunderstanding would in turn influence recruitment of patients to the project: ‘*there’s some things which we realised we had to explain to the public because without which we couldn’t do the project*’ (Interviewee 17). As such, participants spoke about the need to ‘monitor perceptions’ (‘*you should monitor it [interactions with the private sector] extremely carefully and in terms of public perception*’ (Interviewee 6)) and ‘earn [the] trust’ of the public by moving slowly towards more controversial issues. So, for example, recognising that the public had concerns about allowing commercial companies to access genomic data Genomics England postponed any major announcements on the matter until they felt that they had earned trust by demonstrating openness and transparency: ‘*it is acknowledged that commercial use of data is controversial and so we have to earn the trust by showing you what isn’t involved*’ (Interviewee 13).

Such narratives highlight the beginnings of the two parallel and potentially conflicting accounts of public engagement which we discussed in the introduction to the article. In one instance, openness and transparency are narrated as intrinsically important to any public engagement strategy working with public money, and in the other instance, openness and transparency are viewed as an extrinsic means to ensure the project’s successful recruitment of patients.

### Public support as a route to success

Similar to the duel effect accounts of openness and transparency described above, two differing narratives also emerged in interviewees’ accounts about the purpose of public engagement more broadly – as both an intrinsically good practice and as a practice with the goal of ensuring patient recruitment.

On one hand, public engagement was perceived as good practice (‘*involvement of patients and the public … is good practice*’ (Interviewee 11), and as an intrinsic ethical good which gave space to the public to learn and debate:

Public engagement is inherently an ethical enterprise in two ways. One is that science is public … I think science is a democratic enterprise and people have a right to know what’s going on and a right to debate what, how and why … The other level is that … some of us would say ethics is exactly the public conversation about what it is that we are trying to achieve and why and who has a say and developing ethical principles can only be done successfully through the building of a public consensus about what they ought to be and what they should say. (Interviewee 13)

On the other hand, interviewees viewed public engagement as a means of fulfilling an extrinsic goal. This goal was the generation of essential public support to ensure the success of the 100,000 Genomes Project (‘*you need the engagement of the people … it’s going to be most successful if people understand about it*’ (Interviewee 11); ‘*if you don’t have the support of the public, then really the project – it is one of those things that could bring this project down*’ (Interviewee 12)). In referring to ‘success’, interviewees were talking about the need to recruit patients (‘*if the public lose confidence in the probity of the venture, then they will refuse to participate*’ (Interviewee 10)). Therefore, as was discussed in relation to openness and transparency, the public engagement strategy became a mode with which recruitment could be achieved:

I think it’s just a case of educating, supporting and getting people on side and … then they’ll be asking questions [if they are not invited to participate in the project]. ‘Well, hang on I’ve got this, why am I not being asked if I want to be part of this 100,000 Genome Project. (Interviewee 11)

Only one interviewee felt that since the genomes project only related to a subset of patients, public engagement at the general population level had little relevance in terms of patient recruitment. This interviewee ‘*doubt[ed] that having a general debate now is really going to be the way to encourage people to give genomics data at the point at which they get their cancer diagnosis*’ (Interviewee 19). Although Interviewee 5 noted that a public engagement strategy directed to those ‘publics’ working within the NHS could ensure the success of the genomes project, along with levels of recruitment: ‘*I think it’s that support all the way down – from the consultant that somebody might see in the hospital – the nurse, the receptionist, everyone is going to have a positive vibe about this to get it going*’ (Interviewee 5).

### Tensions between intrinsic and extrinsic notions of public engagement

Our data suggest that the need for public support and the need for patient recruitment was so vital to Genomics England that public engagement strategies aiming to fulfil this purpose often overshadowed the notion of public engagement as being an intrinsically good practice directed solely at encouraging public debate and increase public understanding. Interviewees’ narratives provided examples of how Genomics England’s approaches to public engagement often emerged from a strategic need for the project to succeed rather than from an intrinsic desire to implement good practice. Furthermore, actions taken on the basis of public consultation and engagement, such as the aforementioned decision to change the approach to storing data in a ‘cloud’, were viewed as an effort by Genomics England to maintain public trust and support. There was an awareness that instigating an ethical public engagement approach was necessary not only as a goal within itself but also to ensure public confidence:

I think by bending over backwards to have public trust at the center of our objectives [is] the right decision for sure. The ways in which we have limited access to the data make it harder to get hold of it; it makes it more difficult for researchers and definitely more difficult for industry. So, all of those compromises, [are] the things that we have accepted in order to sustain trust. (Interviewee 17)

Interviewees’ narratives which drew on ‘ethics’ and ‘good practice’ as an intrinsic rationale for engaging with the public often reverted to this need for public support (‘*it’s important that we’re not just ethical but that we’re seen to be ethical as well to provide public confidence … I think that that is a necessity of what we need for public engagement*’ (Interviewee 2). One can see the importance of public support to Genomics England as a brand in other decisions that were taken for and by the organisation. For example, the decision to situate its main activity within the NHS which is seen as a trustworthy institution (Hazelton and Petch, 2015): ‘*in some ways having too strong a Government interest in this is not necessarily a good thing. So they’re keeping the brand genomics England slightly separate … And also keeping it, like I say, aligned to the NHS brand*’.

By allowing public engagement to have the dual role as a means to gain public support meant that the lines between informing and encouraging debate on one hand, and engaging people for recruitment purposes on the other hand, sometimes became blurred. There sometimes seemed to be a fine line between the notion of ‘selling’ the project to generate engagement and support and of ‘overselling’ the project, both of which might overshadow the more objective desire to inform and engage as more traditionally understood. Interviewees’ narratives diverged in terms of their own personal perspectives on the balance between telling, selling and overselling. All interviewees talked about the genomics project ‘being sold to the public’ and as being ‘sellable’ (‘*at the moment it’s been sold to the public …*’ (Interviewee 9); ‘*probably what is sellable is the fact that … it will improve their health care*’ (Interviewee 2)). There was no problem inherent in selling a project which they all felt was valuable, well conceived and necessary within the context of a twenty-first-century health service (‘*the project is a really good decision*’ (Interviewee 18). However, while most did not view this selling as *over*selling (‘*we mustn’t oversell this project … and I think that’s pretty much done*’ (Interviewee 5); ‘*there hasn’t been a major big campaign*’ (Interviewee 19)), other interviewees were less convinced, noting a tendency for Genomics England to overpromise the project’s benefits: ‘*I do wonder whether at times it was oversold and over promised*’ (Interviewee 8); *‘I think they’re a bit over on the hype*’ (Interviewee 9). Indeed, Interviewee 16, when asked about engagement, spoke about elements of a public-relations (PR) campaign showcasing Genomics England’s success stories:

they’ve made videos. When we ask them would they allow us to show pictures of them when we present, they say absolutely; we want people to see us because we want others to know that there is hope for them to get answers from the programme.

The blurred boundaries between engaging, selling and overselling did not go unnoticed by interviewees, some of whom felt Genomics England’s engagement strategy sometimes lent too much towards the goal of building a ‘climate of acceptance’ to drive recruitment. For example, in the extract below, Interviewee 13 criticises those individuals at Genomics England who view public engagement in terms of the old-fashioned public understanding of science model, and as a mode to garner support from the public:

Some people think it’s [public engagement] purely about building a climate of acceptance. That it’s about reassuring people that this is useful, that the details are complex but they’re basically sound and please let us get on with it. Some people are wedded to the more old fashioned public understanding of science model. (Interviewee 13)

This view, that is, that public engagement is about presenting information about genomics in the ‘right’ way to gain support for recruitment, was corroborated by Interviewee 18: ‘*[some of the strategies imply that] if you present stuff to people in the right way, it will convert them*’. Similarly, Interviewee 2 took the view that trying to ‘persuade’ the public of the importance of the genomes project was problematic as a goal of the public engagement strategy: ‘*I do sometimes worry that we want to try and persuade the whole population that this is a good thing, whereas actually we shouldn’t be doing that*’.

## 4. Discussion

Our findings have highlighted a theory–practice gap in public engagement and the potentially conflicting roles that emerged within Genomics England’s public engagement strategy. On one hand, interviewees narrated an account which respects public engagement as being intrinsically important, and as being ‘good practice’ in terms of informing the public, and allowing the public to raise any potential concerns about the project and then make their own decisions on the basis of the understanding generated by an ‘objective’ presentation of the ‘facts’. On the other hand, there was recognition of the substantial extrinsic value to public engagement in terms of generating public support, and ultimately encouraging recruitment to the project. Moreover, our interviewees provided a range of examples in which the clinical (and political) need to demonstrate movement towards the publicly stated goal of mapping 100,000 genomes meant the need to actively generate public support overshadowed much of the public engagement agenda. At some level, this issue is further complicated by the interesting hybrid identity of the 100,000 Genome Project as a clinical and research endeavour. When considering the project as a clinical entity, it is relatively unproblematic to think of Genomics England alerting cancer and rare disease patients to the opportunity of engaging with a new level of care, accentuating the possibility of this contributing to their pursuit of a diagnosis and/or effective treatment. However, when seen as a research project, recruitment to the 100,000 Genome Project should ideally take place against a background of researcher equipoise (Freedman, 1987). That is, at the level of public engagement, potential participants should be given a clear sense of the newness, uncertainty and lack of existing knowledge about the project, and how the choice to participate will benefit the patient in terms of their clinical experience.^6^ We would argue that the need to own and negotiate this potential conflict is the key responsibility of Genomics England when conceiving and implementing their public engagement strategy. This is not a unique challenge, with similar examples having been reported previously (Pieczka and Escobar, 2013; Van Bekkum et al., 2016).

In terms of Genomics England, we would also argue that it is unrealistic and possibly even inappropriate to expect the organisation to keep the momentum going on a more generalised and potentially critical debate on the value or otherwise of genomic medicine. An earlier phase of public engagement, in which Genomics England was not necessarily directly involved, had already promoted public debate around the value of genomic medicine, noted the objective goods of the project and generated enough support for politicians to push the project forward.^7^ Thus, Genomics England was able to move forward on the basis that the initial goals of public engagement had been achieved and the need for them to have a discussion with the public about the value of the 100,000 Genomes Project was no longer required. This is not to say that Genomics England ignored or deflected the issues that might have arisen in such a debate, just that it was not seen as core business to continue ‘discussion for discussion’s sake’, their response was one of problem solving and practical mitigation of concerns. Genomics England was therefore able to start its public engagement strategy from a position where their mission already had political and expert professional support and endorsement, and was perceived to have social value. It would be naive to assume that support or indeed knowledge of the project was widespread within the professional groups which would ultimately be involved in its research. But the preceding debate and level of acceptance at the highest levels gave moral permission for interviewees to commence their engagements with wider professional groups and the public with the underlying assumption that the genomes project was a good idea and had social worth.

The purpose of engagement then became informing, and listening to, the public against an already positive backdrop to fine-tune and ultimately introduce the programme of research and clinical intervention. This aligned with the extrinsic purpose of public engagement to ‘sell’ the benefits of the project and gain public support for recruitment. As such, interviewees were convinced that pursuing both goals of public engagement gave their strategy both intrinsic (in terms of listening to the public and helping them navigate the project’s complex associated issues) and extrinsic (in terms of generating support and ultimate recruitment) social worth and were therefore morally permissible.

While interviewees seemed convinced of the moral permissibility of the public engagement strategies employed by Genomics England, it is important to acknowledge the difficult tensions that were inevitably produced between ‘selling’ the project and trying not to ‘over sell’. We argue that Genomics England must remain vigilant about ensuring any strategies to increase public support do not amount to overselling the programme to those who might be recruited as participants now or in the future, thereby respecting the need for balance and equipoise. The danger is that if an appropriate balance cannot be reached between selling and overselling, managing expectations and supporting rational decision-making in clinical practice will become a serious ethical and practical issue. Consideration of this issue can be informed by recent work in other areas of medicine. For example, in the field of brain stimulation for children and young people with movement disorders, hopeful patients and their families often arrive at clinics with highly optimistic and hyped visions of what the brain stimulation technology can deliver. Such visions are fuelled from promotion material within the media, often disseminated by clinicians themselves as they endeavour to promote their research and its benefits (Brown and Michael, 2003). Within the clinic, health professionals must then manage patient expectations by deploying less-optimistic, uncertain and modest visions of the technology and the patient’s future (Gardner et al., 2015). A more positive example is possibly the soon-to-be attempted mitochondrial transfer procedure where the introduction of a cutting-edge procedure has been preceded by a long-running and nuanced public debate which has allowed clinicians and potential patients to manage and recalibrate their expectations in line with scientific evaluation.

Managing the expectations attached to the 100,000 Genomes Project is further complicated by the blurred boundaries created by the project’s dual research-clinical hybrid nature. Such blurred boundaries might make it difficult for patients/families to understand the project’s purpose and rationale fully, and if expectations are oversold (especially the clinical benefit aspect of the project compared to its research aspect), patients/families might expect some immediate clinical benefit, such as an effective treatment, especially because sequencing was performed as part of a clinical service, on NHS premises, by NHS staff and sometimes following other clinical tests (Dheensa et al., forthcoming). We have discussed this issue at length elsewhere, where we suggest that managing such expectations (as well as other issues concerns related to the research-clinical hybrid nature of the project) would best be negotiated by re-conceptualising the distinction between research and clinical practice (Dheensa et al., forthcoming).

For Genomics England, it is important to acknowledge the relatively low levels of public understanding about the 100,000 Genome Project which will unfold within the NHS. While it will remain an important element of their work to engage with and recruit the professionals who will need to be on board to deliver the service and conduct the research, if their public engagement strategy moves too far towards overselling to this group, it may in turn lead to heightened patient and public expectations about the clinical benefits of the genomes project. These expectations about the project then place pressure on clinicians, who need to manage them at the level of clinical practice.

Looking forward, it is crucial that those charged with ‘selling’ the genomic agenda need to do so in a manner that permits potential participants to understand the balance of costs and benefits, weigh them appropriately and manage their expectations appropriately as they embark upon their journey as a patient/participant in a complex and innovative intervention. This needs to be done in a setting which allows open space within which dialogue between Genomics England and potential patients/patients is nurtured and encouraged, a way in which Genomics England acts in a non-authoritarian manner in spite of the need to drive recruitment, and in a way which allows ‘trust’ to permeate the relationship (Bowman, 2017). As such, in terms of recommendations, we simply draw attention to the possibility of a conflict of interest and ask that Genomics England be mindful of this, and of the generic nature of public engagement versus the more specific engagement required to attract people to participate in research. Such recommendations are especially important as we move further towards the UK Chief Medical Officer’s *Generation Genome* – a vision to bring genomics into all aspects of NHS care – and a venture that will require a strong public engagement strategy which recognises and remains vigilant of the theory–practice gap in public engagement initiatives as it proceeds forward to the intersection of research and clinical care (Davies, 2017).

## Notes

1.
http://www.publications.parliament.uk/pa/ld200809/ldselect/ldsctech/107/10711.htm

2. Other research-hybrid projects not on a national scale include, for example, the US Geisinger health systems’ MyCode^®^ genomics data system and biobank, and the US Mayo Clinic’s Center for Individualized Medicine. The research-hybrid set-up is interesting in terms of novel conceptions of innovation pathways and is the subject of a forthcoming article (Dheensa and Samuel).

3.
https://www.genomicsengland.co.uk/about-genomics-england/

4. There are 13 National Health Service (NHS) Genomics Medicine Centres (GMCs), each of which are centres of genomics excellence. They are responsible for delivering the genomes project in terms of patient recruitment and consent, and DNA collection, extraction and transport for sequencing.

5. We note that the opinions of those whom placed intrinsic importance in openness/transparency most likely stem from a social constructed backdrop of society becoming more open and transparent in order to build trust between experts and the ‘public’ (as Moore (2010) notes, ‘*a deficit in scientific understanding is being replaced by talk of a deficit in trust … the trust deficit is to be remedied by openness, consultation and dialogue with the public*’. As such, the intrinsic and extrinsic views of openness and transparency are blurred since, as we show below, the need to recruit patients also relates to the perceived need of gaining public trust. Nonetheless, the two separate perspectives were apparent in interviewees’ narratives, and at the very least represent differences in priorities – one trying to adhere to the intrinsic importance of openness (even if such principles are situated/constructed) and the other focused on the need to recruit patients.

6. Here, we are not suggesting this does not occur at the level of patient consultation. Just that for research projects, it is ordinarily part of any public engagement strategy.

7. We are not arguing the value or worth of the 100,000 Genomes Project here, since this is beyond the premise of the article. Rather, we are illustrating how political endorsement gave the project a perceived worth.
